# Surgery vs. Radiosurgery for Patients with Localized Metastatic Brain Disease: A Systematic Review with Meta-Analysis of Randomized Controlled Trials

**DOI:** 10.3390/cancers15153802

**Published:** 2023-07-26

**Authors:** Giorgio Fiore, Leonardo Tariciotti, Giulio Andrea Bertani, Dario Gagliano, Antonio D’Ammando, Antonella Maria Ampollini, Luigi Schisano, Stefano Borsa, Mauro Pluderi, Marco Locatelli, Manuela Caroli

**Affiliations:** 1 Unit of Neurosurgery, IRCCS Ca’ Granda Foundation Ospedale Maggiore Policlinico, Via Francesco Sforza, 35, 20122 Milan, Italy; 2 Department of Medical and Surgical Pathophysiology and Transplantation, University of Milan, 20122 Milan, Italy; 3 Department of Neurosurgery, National Hospital for Neurology and Neurosurgery, Queen Square, London WC1N 3BG, UK

**Keywords:** brain metastasis, radiosurgery, surgery, surgical resection, radiotherapy, oligometastasis, solitary brain metastasis, whole brain radiotherapy, survival, progression-free survival

## Abstract

**Simple Summary:**

With an incidence of 14 cases per 100,000 people per year, brain metastases (BMs) are the most frequent malignant intracranial lesions, occurring in up to 40% of all patients with solid tumors. Median survival with the best standard of care is between 8 and 16 months, but subcohorts of patients achieved longer survival rates. These subcohorts include patients with good performance status and single (or very few) BMs eligible for aggressive treatments, namely surgical resection and stereotactic radiosurgery (RS). There is a lack of evidence to support the superiority of surgery or RS as first-line treatment in solitary and oligometastatic brain disease, since previous attempts to systematically review the corpus of evidence have failed to produce any conclusive findings. This systematic review with meta-analysis aims to provide a quantitative synthesis of the results of RCTs investigating the efficacy and safety of surgery compared to RS, combined or not with WBRT, for localized metastatic brain disease.

**Abstract:**

**Purpose:** To analyze the efficacy and safety of surgery compared to radiosurgery (RS), combined or not with whole brain radiotherapy (WBRT), for localized metastatic brain disease. **Methods:** A systematic review with meta-analysis was conducted following the Preferred Reporting Items for Systematic Reviews and Meta-Analyses (PRISMA) 2020 guidelines. The inclusion criteria were limited to randomized controlled trials (RCTs) that compared surgery and RS for patients with up to 3 metastases (median diameter ≤ 4 cm). The primary outcomes were represented by overall survival (OS) and local brain progression-free survival (PFS), with the rate of complications as a secondary outcome. The pooled estimates were calculated using random forest models. The risk of bias was evaluated using the RoB2 revised tool and the certainty of the evidence was assessed according to the GRADE guidelines. **Results:** In total, 11,256 records were identified through database and register searches. After study selection, 3 RCTs and 353 patients were included in the quantitative synthesis. Surgery and RS represented the main intervention arms in all the included RCTs. **Conclusions:** A low level of evidence suggests that RS alone and surgery followed by WBRT provide an equal rate of local brain PFS in patients with localized metastatic brain disease. There is a very low level of evidence that surgery and RS as main interventions offer equivalent OS in the population investigated. A reliable assessment of the complication rates among surgery and RS was not achievable. The lack of high-certainty evidence either for superiority or equivalence of these treatments emphasizes the need for further, more accurate, RCTs comparing surgery and RS as local treatment in patients with oligometastatic brain disease.

## 1. Introduction

With an incidence of 14 cases per 100,000 people per year, brain metastases (BM) are the most frequent malignant intracranial lesions, occurring in up to 40% of all patients with solid tumors and representing a significant cause of severe morbidity and mortality [1]. Despite recent advances and several clinical trials, some issues in their management remain unresolved [2]. Median survival with the best standard of care is between 8 and 16 months, but subcohorts of patients achieved longer survival rates [3]. They include patients with good performance status and single (or very few) BMs eligible for aggressive treatments, namely surgical resection and stereotactic radiosurgery (RS) [4]. The combination of these interventions, with or without systemic chemotherapy, may prolong survival rates and disease-free intervals up to two years, with a small proportion of patients alive at five years [5,6,7].

Several clinical trials have investigated the effectiveness of surgery and radiosurgery, with or without adjuvant WBRT, as first-line treatment for metastatic patients with preserved functional status; however, it is still unclear whether appropriate patients harboring oligometastatic brain and controlled systemic disease might respond differently to surgery or RS [2]. According to previous results, surgery and RS represent equivalent interventions in terms of safety, both being responsible for grade 3–4 toxicity events in less than 5% of patients, with no significant differences. The reported survival differences attributable to aggressive local intervention remain controversial: while Patchell et al. reported a positive impact of surgery followed by WBRT vs. WBRT alone, other authors have described limited or absent beneficial effects of radical surgery for solitary BMs [8,9,10,11].

Assessing whether surgery and RS provide similar oncological outcomes is a challenging question to address in randomized settings. The comparison of these two treatments might be relevant to only a small proportion of patients encountered in oncological practice, as very few are eligible for both surgery and RS, making patient recruitment difficult. Patients’ preference for avoiding invasive procedures and general anesthesia, or in favor of a more radical approach, presents additional issues to consider.

No evidence has so far supported the superiority of surgery or RS as first-line treatment in solitary and oligometastatic brain disease, since previous attempts to systematically review the corpus of evidence have failed to elaborate any conclusive findings [2,12]. The specific difficulties are high heterogeneity of primary tumor histological types, patients’ functional status, divergences in the administered adjuvant and neoadjuvant treatments. To control the unavoidable variance related to these factors, secondary evidence based on RCTs might provide higher-certainty results compared to those from observational studies.

This systematic review with meta-analysis aims to provide a quantitative synthesis of the results of RCTs investigating the efficacy and safety of surgery compared to RS for localized metastatic brain disease. The rationale for this study is to understand whether surgical resection, in addition to radiotherapy, RS or WBRT, can improve overall survival and disease recurrence in this patient population compared to a less invasive radiosurgery approach.

## 2. Methods

A systematic review with meta-analysis was conducted according to the Preferred Reporting Items for Systematic Reviews and Meta-Analyses (PRISMA) 2020 guidelines [13].

### 2.1. Type of Studies

We included randomized controlled trials (RCTs) that compared surgery and radiosurgery for the treatment of patients with localized metastatic brain disease. Observational studies, case series and case reports were excluded from the current investigation to reduce inaccuracy related to different research settings and the high risk of biasing inclusion criteria.

### 2.2. Population

Adult patients (aged 18 or over) were included in the current investigation if they presented with the following:

- three or fewer brain metastases with a median diameter equal to or less than 4 cm (localized metastatic brain disease);
- primary tumor histological diagnosis;
- negative history of previous cranial focal treatments.

### 2.3. Type of Interventions

RCTs were included if they compared the main outcomes of patients undergoing surgical resection and radiosurgery. Any stereotactic radiation therapy was included: gamma knife (GK), linear accelerators (LINAC), hypo-fractionated and fractionated radiation therapy, etc. RCTs were included even if whole brain radiotherapy (WBRT) was administered as adjuvant treatment, as long as they compared surgical resection and radiosurgery. Both chemotherapy and target therapies were accepted as co-interventions.

### 2.4. Primary Outcome Measures

The primary outcome measures were represented by the overall survival (OS) and the local (cranial) progression-free survival (PFS) at treated sites. Death and local recurrence rates at 1, 2 and 3 years were also identified as primary outcomes.

### 2.5. Secondary Outcome Measures

The rate of medical complications in the two intervention groups constituted the secondary outcome of this meta-analysis. We looked at any neurological complications, such as local neurological deficits, hearing loss, tiredness, etc. Any local side effects related to the administered intervention were also investigated (e.g., nausea, vomiting, skin complications, infection of the wound, post-surgical or post-radiation hematomas, etc.).

### 2.6. Information Sources and Search Strategy

The following databases and registers were selected as information sources: PubMed, CENTRAL and ClinicalTrials.gov. The first search was concluded in March 2022. An updated search was conducted in January 2023. A third search was carried out by exploring the included RCTs’ reference lists and the studies citing them. The following research terms were used to identify the relevant references: cerebral, intracranial, brain, metastases, and surgery, along with their MeSH terms. The initial search strategy is available in the Supplementary Material File S1. No filters were applied during the research process. We included only studies whose results were reported in English.

### 2.7. Selection Process

Four authors (G.F., L.T., A.D.A. and D.G.) independently reviewed the titles and abstracts of the retrieved articles, classifying them as included, excluded and possible. During this stage, articles that did not meet the inclusion criteria were excluded (such as reports not written in English, studies on animals, studies in vitro, reviews, commentaries, case reports, etc.). In case of disagreement between two or more authors, the consensus was reached by jointly conducted examination of the full text. Afterward, the full texts of the articles that were initially classified as included or possible were independently assessed by the authors (G.F., L.T., A.D.A. and D.G.). Again, in case of disagreement, a consensus was reached by discussion. No automatic tools were employed during the selection process.

### 2.8. Data Collection

Two reviewers (G.F. and A.D.A.) independently collected the data from the included RCTs. A time-to-event survival analysis was conducted for the primary outcomes OS and PFS, and the intervention effect was expressed as hazard ratio (HR). The death and local recurrence rates at 1, 2 and 3 years were analyzed as dichotomous data, and the intervention effect was expressed as a risk ratio (RR). Likewise, the rate of medical complications in the two intervention groups was analyzed as dichotomous data and expressed as a RR. If the HR for the survival outcomes was not directly reported in the included RCTs, it was calculated using the methods provided by Tierney et al. [14] or suggested in chapter 6.8.2 of the Cochrane Handbook for Systematic Reviews of Interventions (Higgins 2022—https://www.training.cochrane.org/handbook (accessed on 1 June 2022)).

### 2.9. Heterogeneity

Clinical heterogeneity was assessed by evaluating the patient population features, the types of intervention, the co-interventions, and the settings. Methodological heterogeneity was assessed by estimating the risk of bias (RoB). Statistical heterogeneity was evaluated using Cochran’s Q (χ^2^) test, *I*^2^ and tau^2^.

### 2.10. Risk of Bias

Two authors (L.T. and G.F.) independently assessed the risk of bias for the included RCTs using the second version of the Cochrane tool for assessing the risk of bias in randomized trials (RoB 2) [15]. The following five specific domains were investigated: (1) bias arising from the randomization process; (2) bias due to deviations from intended interventions; (3) bias due to missing outcome data; (4) bias in the measurement of the outcome; (5) bias in the selection of the reported result. Any discrepancies were resolved by discussion. The study was judged to be at low risk of bias if all the domains matched this result. The study was considered to have some concerns if at least one of the domains was assessed to have concerns. Finally, the study was judged at high risk of bias if at least one domain matched this result or if more domains raised concerns that lowered the confidence in the result.

### 2.11. Reporting of Bias Assessment

We planned to assess the publication bias by inspecting the funnel plots if more than five studies contributed to the pooled estimates (see GRADE guidelines—https://www.gradeworkinggroup.org (accessed on 1 June 2023)). To reduce the risk of reporting bias related to missed small studies, the authors searched the main databases and registers as well as the reference list of all included RCTs. The risk related to lag biases was also investigated.

### 2.12. Synthesis Methods

A pooled estimate was calculated if at least two studies were available for the meta-analysis. A generic inverse-variance method was employed to pool the HRs and confidence intervals (CIs) of the primary time-to-event (OS and PFS) outcomes. We pooled the HRs if the time-to-event data were extracted through the Kaplan–Meier method. The exact Mantel–Haenszel method without continuity corrections [16] was used to pool the RRs and CIs of the dichotomous primary outcomes (death and local recurrence rates) and secondary outcomes (incidence of complications). A random-forest model was employed to address both sampling error and other sources of variance. The between-studies heterogeneity (*t*^2^) was assessed using the Paule–Mandel (PM) method [17]. We planned to employ Knapp–Hartung (KH) adjustments to calculate the CIs around the pooled estimates in the case of nonhomogeneous effects [18]. This meta-analysis was performed using R software 4.2.2 (R Foundation for Statistical Computing, Vienna, Austria; http://www.r-project.org/index.html (accessed on 1 September 2022)). Forest plots were used to display the pooled effects, the CIs, the weight of the included RCTs, and the estimated measures of inter-study heterogeneity.

### 2.13. Subgroup Analysis

The following subgroup analyses were planned:

- number of intracranial metastases (one vs. more than one intracranial metastasis);
- primary tumor histological type;
- co-interventions (e.g., whether WBRT was administered after the primary interventions);
- RPA or KPS class.

### 2.14. Sensitivity Analysis

Sensitivity analyses were conducted in the case of one or more studies with a high risk of bias being included in the pooled estimate.

### 2.15. Certainty Assessment

The certainty of the evidence was jointly assessed by two authors (G.F. and L.T.) according to the GRADE guidelines [19]. The following domains were used for downgrading the quality of evidence: (1) limitations in study design and execution (risk of bias), (2) inconsistency of results, (3) indirectness of evidence, (4) imprecision and (5) publication bias. The quality of the evidence was upgraded according to the following factors: (1) the large magnitude of the effect, (2) the dose–response gradient and (3) the plausible confounding effect. Accordingly, the certainty of the evidence was graded as high, moderate, low, or very low according to GRADE guidelines [20]. ‘Summary of findings’ tables were built using the GRADEpro GDT software (https://www.gradepro.org (accessed on 1 June 2023)).

## 3. Results

**Figure 1:**
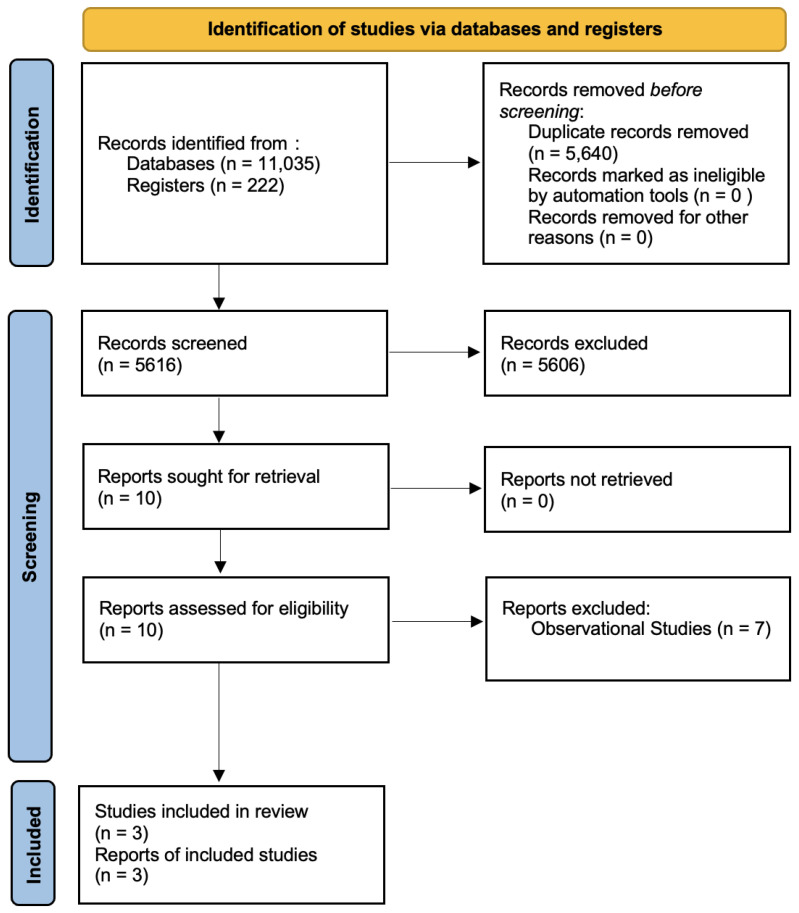
Flow diagram according to the PRISMA guidelines.

Our initial search led to the identification of 11,035 database references and 222 register references, with a total of 11,256 references being included in the first screening. After the removal of duplicates, 5616 references were screened by title and abstract. Ten studies were evaluated for eligibility by assessing the full texts. Seven studies were excluded because they were determined to be observational [21,22,23,24,25,26,27]. In total, 3 studies and 353 patients were included in the meta-analysis [28,29,30]. The flow diagram following the PRISMA guidelines is shown in Figure 1.

### 3.1. Studies

All included RCTs were published as journal articles. The most represented primary tumor was lung cancer (209 patients, 59%), followed by colorectal cancer (47 patients, 13%) and breast cancer (44 patients, 12%). The median patient age was 60 years, and 137 of the patients were female (39%). The majority of patients had solitary brain metastases (311, 88%). No patients with more than two metastases were included in this meta-analysis. The median diameter was 20 mm (4–40 mm). The median Karnofsky performance status was 80, and the most frequent recursive partitioning analyses (RPA) class was 1 (174 patients, 49%). Radiosurgery and surgical resection represented the main intervention arms in all the included RCTs, with or without adjuvant WBRT. Radiosurgery was administered as GK in Muacevic et al., LINAC in Roos et al., and both GK and LINAC in the EORTC 22952-26001 study. The mean dose varied between 21 and 25 Gy for GK and between 15 and 20 Gy for LINAC. WBRT was administered at a dose of 2 Gy ×20 in Muacevic et al. and 3 Gy ×10 in the other two included studies.

### 3.2. Risk of Bias

**Figure 2:**
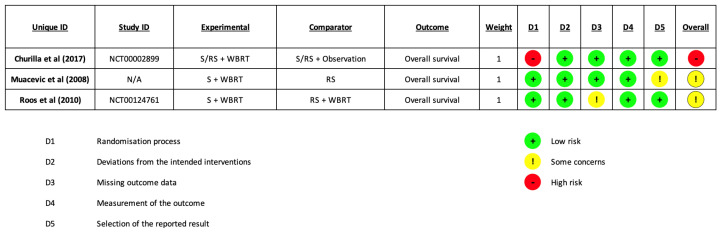
Risk of Bias [28,29,30].

Risk of bias assessment was performed for each included study during data extraction. See Figure 2 for a graphical summary. Additional information about per-trial evaluations are available in Supplementary Table S1. The overall RoB was judged “high” for Chirulla et al. [28] and as having “some concerns” for Muacevic et al. [30] and Roos et al. [29].

### 3.3. Effects of Interventions

#### 3.3.1. Primary Outcomes

##### Overall Survival

**Figure 3:**
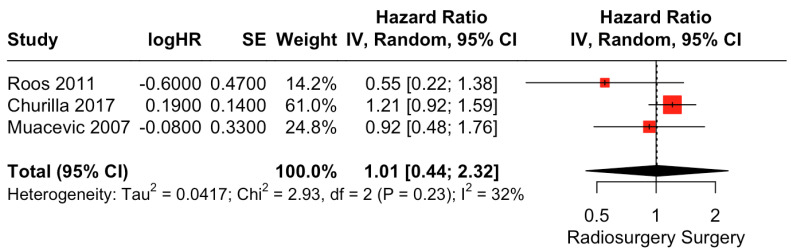
Forest plot of OS comparison between radiosurgery and surgery [28,29,30].

There was no clear difference in OS between surgical resection and radiosurgery. The pooled estimate (HR) was 1.01 (CIs: 0.44; 2.32; *I*^2^ = 32%; *p* = 0.96; Figure 3).

**Figure 4:**
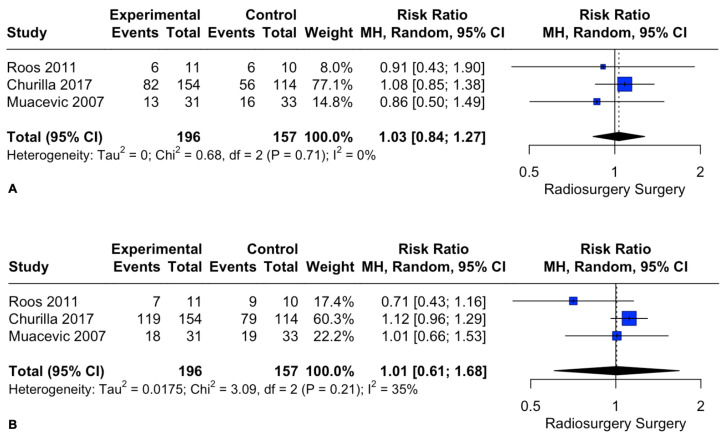
Forest plot of death rate comparison between radiosurgery and surgery at 1 (**A**) and 2 (**B**) years [28,29,30].

Similarly, patient death rates at 1 and 2 years did not differ between the two intervention groups. The pooled estimates (RR) were 1.03 (CIs: 0.84; 1.27; *I*^2^ = 0%; *p* = 0.76) and 1.01 (CIs: 0.61; 1.68; *I*^2^ = 35%; *p* = 0.96), respectively (Figure 4).

##### Subgroup Analysis

No subgroup analyses were performed, as none of the included studies explored the impact of the different numbers of metastases, primary tumor histology, different types of co-intervention, or patient functional classes.

##### Progression-Free Survival

**Figure 5:**
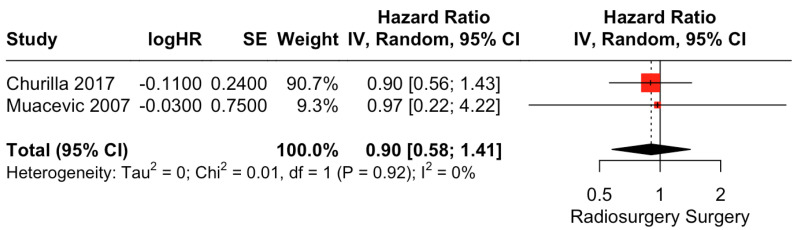
Forest plot of PFS comparison between radiosurgery and surgery [28,30].

There was no clear difference in PFS between surgical resection and radiosurgery. The pooled estimate (HR) was 0.90 (CIs: 0.58; 1.41; *I*^2^ = 0%; *p* = 0.65; Figure 5).

**Figure 6:**
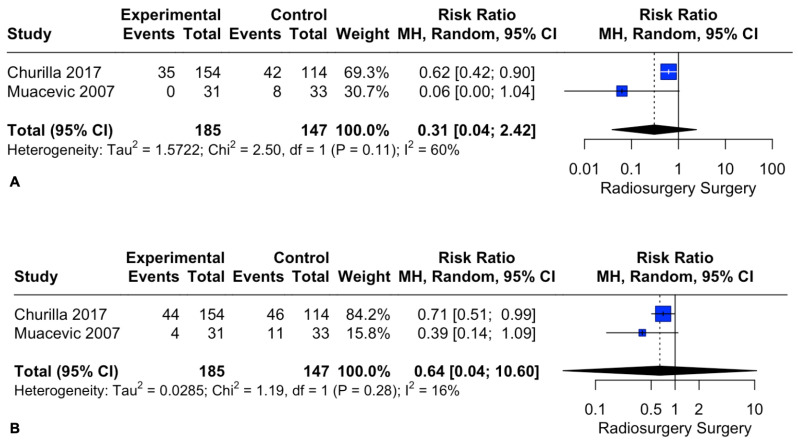
Forest plot of recurrence rate comparison between radiosurgery and surgery at 1 (**A**) and 2 (**B**) years [28,30].

Patient local recurrence rates at 1 and 2 years did not differ between the two intervention groups. The pooled estimates (RR) were 0.31 (CIs: 0.04; 2.42; *I*^2^ = 60%; *p* = 0.26) and 0.64 (CIs: 0.04; 10.6; *I*^2^ = 16%; *p* = 0.3), respectively (Figure 6).

Since Roos et al. [29] included in the same analysis the disease relapse at treated sites and the occurrence of new brain metastases, we were unable to include this study in the pooled analysis for local PFS and local recurrence rates at 1 and 2 years.

##### Subgroup Analysis

No subgroup analyses were performed for different numbers of metastases, primary tumor histology, or patient functional classes, since none of the included RCTs analyzed the impact of these factors independently.

**Figure 7:**
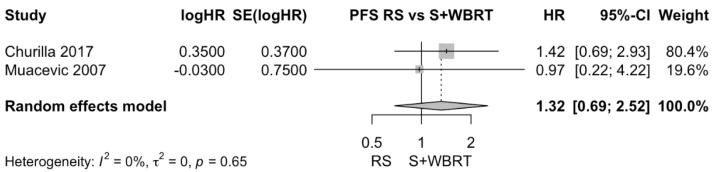
Forest plot of PFS comparison between radiosurgery alone and surgical resection + WBRT [28,30].

Churilla et al. performed distinct analyses exploring the adjunctive effect of WBRT on local PFS after both surgery and RS [28]. Therefore, subgroup analyses of radiosurgery alone vs. surgery + WBRT were conducted for local PFS. There was no clear difference in local PFS between radiosurgery alone and surgical resection + WBRT. The pooled estimate (HR) was 1.32 (CIs: 0.69; 2.52; *I*^2^ = 0%; *p* = 0.4; Figure 7).

#### 3.3.2. Secondary Outcomes (Complication Rates)

We were unable to pool the RR of complications between radiosurgery and surgical resection, as different methods and measures were used to assess and report these outcomes. Muacevic et al. found that acute and late grade 1 and 2 complications were more frequent in the intervention group of surgery + WBRT compared to radiosurgery alone. In contrast, no significant differences were found for early or late grade 3 and 4 complications. The authors reported a higher rate of intratumoral bleeding and seizures in the radiosurgery group [30]. Roos et al. reported no statistically significant differences in acute and late treatment-related toxicity of any grade [29]. The EORTC 22952-26001 study provided a complication rate comparison between surgery and radiosurgery only for adverse skin events, with no statistical differences between the two intervention arms [28].

### 3.4. Sensitivity Analysis

We determined a study to have an overall high risk of bias if it was judged to have a high risk of bias in at least one of the five domains. One study met this criterion [28], and sensitivity analyses excluding this study were performed for OS and death rates at 1 and 2 years.

**Figure 8:**
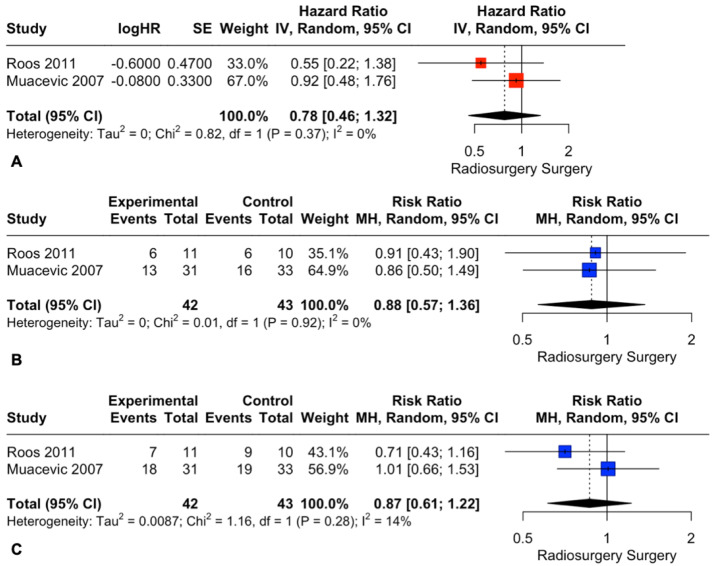
Forest plots of OS (**A**) and death rate comparison at 1 (**B**) and 2 (**C**) years between radiosurgery and surgery [29,30].

Exclusion of the study with a high risk of bias did not change the pooled estimates for OS (HR: 0.78; 95% CI: 0.46, 1.32, I2 = 0%; *p* = 0.35, Figure 8A) or death rates at 1 and 2 years (RR: 0.88; 95% CI: 0.57, 1.36, I2 = 0%; *p* = 0.57 and RR: 0.87; 95% CI: 0.61, 1.22, I2 = 14%; *p* = 0.41, Figure 8B and Figure 8C, respectively).

## 4. Discussion

The treatment of metastatic brain disease has been subjected to relevant improvements in terms of survival outcomes over time. Until recently, the diagnosis of brain metastasis was considered a sign of poor prognosis [31]. Nowadays, the adjunctive value of new target therapies for the treatment of localized metastatic brain disease, as well as the availability of tailored approaches to preserve patients’ quality of life, has led to increased survival rates [3]. As survival outcomes increase, patient expectations regarding their quality of life grow accordingly, fueling discussions about the best local treatment physicians can provide to preserve their function.

### 4.1. Quality of the Evidence

#### 4.1.1. Overall Survival and Death Rates

The present meta-analysis failed to identify significant differences in the overall survival of patients with localized metastatic brain disease undergoing RS or surgical resection as the main local treatment. Nonetheless, the certainty level of the evidence was downgraded to very low because of the indirectness related to the different employment of WBRT as co-intervention, the imprecision related to the small underpowering sample size, and the high risk of bias. Likewise, a very low level of certainty suggested that radiosurgery and surgical resection did not differ in the death rates at 1 and 2 years.

#### 4.1.2. Local Progression-Free Survival and Recurrence Rates

The overall local progression-free survival did not show significant differences between radiosurgery and surgical resection. This result was supported by a very low grade of certainty, attributed to the indirectness due to the inconstant use of WBRT as co-intervention, the imprecision related to the underpowering small sample size, and the high risk of bias. In the same way, there is a very low level of evidence that the local recurrence rates at 1 and 2 years did not differ between the two intervention groups.

A low level of evidence suggests that radiosurgery alone or surgical resection followed by WBRT have similar outcomes in terms of PFS. The low level of evidence considered the imprecision related to the small sample size and the high risk of bias.

#### 4.1.3. Complication Rates

This meta-analysis failed to provide a pooled estimate for the rate of complications among radiosurgery and surgery, mainly because of the different methods that the authors used for reporting this outcome. Of note, all RCTs declared statistically similar incidences of acute and late severe (grade 3 and 4) complications among the two intervention groups.

A “Summary of Findings” table is available as Table S2.

### 4.2. Applicability and Relevance of the Evidence

Despite the prevalence of localized metastatic brain disease in neuro-oncological and neurosurgical practice, this meta-analysis highlights a worrisome lack of high-certainty evidence on this topic. The limited number of available RCTs and the high heterogeneity of co-interventions, primary histological types, and patient functional classes call for further and more accurate RCTs. We found a low level of evidence suggesting that RS alone and surgery followed by WBRT may provide an equal local PFS. This finding replicates the previous results of Liu et al., who conducted a quantitative synthesis with level-III evidence that was flawed by including retrospective and prospective observational studies and insufficiently strict inclusion criteria [32]. Furthermore, our study found no differences in OS among RS and surgical resection groups. Even if the level of certainty was graded as very low for this outcome, the results show consistency with previous reports [21,22,26,29,30,33]. Dasgupta et al. [34] analyzed clinicopathological features and treatments of long-term survivors (patients with metastatic brain disease and OS equal to or greater than 3 years, range of 36–181 months). The authors reported an equal contribution of surgery and radiosurgery to the survival outcomes of these patients (40% and 45% of patients, respectively). Similarly, Punchak et al. investigated the role of surgery and radiosurgery in the context of multiple metastases with a predominant brain lesion. The authors found similar survival rates among the two treatment groups, concluding that adequate treatment should be suggested by patient and tumor features [35]. Finally, a reliable assessment of the evidence concerning the rate of complications among radiosurgery and surgery could not be performed. Higher consistency in the selected reporting methods would be worthwhile in future RCTs.

## 5. Limitations and Future Directions

Although this study was conducted following the PRISMA 2020 guidelines and the data extraction on initial 11,256 references was carried out by four independent reviewers, the main limitation of the results coming from this meta-analysis is represented by the imprecision related to the limited number of identified RCTs. The studies conducted by Roos et al. [29] and by Muacevic et al. [30] were stopped early due to slow accrual, pointing out the difficulty in recruiting patients with localized metastatic brain disease in a randomized and controlled setting. The study conducted by Churilla et al. was judged to be at a high risk of bias, since patients were randomized for WBRT and stratified according to RS and surgery in a secondary analysis [28]. Moreover, despite our efforts to comprehensively combine different treatment strategies comparing radiosurgery and surgery, some emerging approaches were still not available for inclusion in the quantitative synthesis. For instance, radiosurgery after resective surgery is rising as a standard treatment for localized metastatic brain disease, as it can guarantee equal survival and local control compared to WBRT while reducing the incidence of patient cognitive decline [36]. WBRT was found to lead to decline in cognitive performance over a long period, while the cognitive side effects of SRS are usually transient [37,38,39]. The main drawbacks of adjuvant RS relate to difficulties in delineating the target volume of the surgical bed, uncertainties related to dose fractioning, dose prescription, and timing of postoperative SRS, as well as the eventual risk of leptomeningeal spread [40]. The study by Li et al. supports the idea that preoperative SRS can overcome these limitations, suggesting the potential for improved outcomes compared to postoperative SRS [41]. The results deriving from the ongoing NCT03750227 and NCT03741673 trials may add further insights about this recent treatment strategy, potentially extending the evidence of this meta-analysis in a future update.

## 6. Authors’ Considerations

There is no high-certainty evidence claiming for the superiority of either surgery or radiosurgery in patients with localized metastatic brain disease, or for equivalence between the two treatments. Therefore, the treatment decision-making process should include an accurate selection of the right candidates for aggressive local treatments, considering patient, tumor, and available service features. Surgical resection of brain metastases may be preferred if prompt relief of symptoms related to tumor mass effect and massive surrounding edema is needed. Further, surgery is indicated when the histological diagnosis of the primary tumor is unknown, or if additional histological and molecular characterization is needed. Weichselbaum and Hellman suggested that the evolution of cancer metastatic capacity has intermediate steps in which the tumor spread is limited to specific organs [42]. In this context, surgery may offer the advantage of identifying, at a molecular level, true oligometastatic disease, which is distinguished by improved survival outcomes after appropriate local and systemic treatments compared to polymetastatic disease [42]. On the other hand, radiosurgery can represent a better treatment choice for frail patients, or those with small and/or deep brain lesions and limited tumor edema or mass effect. The combination of surgery and radiosurgery, which is currently under investigation in RCT settings, is promising in terms of reduced local recurrence and overall survival rates, and it might represent a successful treatment option for patients with localized metastatic brain disease. When WBRT is chosen as an adjuvant treatment, its long-term cognitive side effects should be discussed during patient counseling.

## 7. Conclusions

There is a low level of evidence that radiosurgery alone and surgery followed by WBRT provide equal local PFS in patients with localized metastatic brain disease. There is a very low level of evidence that surgery and radiosurgery as main interventions offer equivalent overall survival in the population investigated. A reliable assessment of the existing evidence related to complication rates among surgery and radiosurgery was not achievable.

The lack of high-certainty evidence for either the superiority or equivalence of surgery compared to RS demonstrates the need for further, more accurate, RCTs. Therefore, surgery and radiosurgery as main local treatments in patients with oligometastatic brain disease should be considered on an individual basis and selected according to patient and tumor features.

## Supplementary Materials

The following supporting information can be downloaded at https://www.mdpi.com/article/10.3390/cancers15153802/s1, File S1. Search Strategy; Table S1: Risk of Bias; Table S2. Radiosurgery compared to Surgery for patients with localized metastatic brain disease.
