# Supplementary material for: Surgery vs. Radiosurgery for Patients with Localized Metastatic Brain Disease: A Systematic Review with Meta-Analysis of Randomized Controlled Trials
Source: Cancers (Basel). 2023 Jul 26;15(15):3802. doi: 10.3390/cancers15153802 (PMC10417431; doi:10.3390/cancers15153802)
Supplement: Supplementary file 1 [file cancers-15-03802-s001.zip › Table S1.pdf]

Table S1: Risk of Bias

**Unique ID**	1	**Study ID**	NCT00002899	**Assessor**	L.T. and G.F.
**Ref or Label**	Churilla et al.	**Aim**	assignment to intervention (the 'intention-to-treat' effect)		
**Experimental**	S/RS + WBRT	**Comparator**	S/RS + Observation	**Source**	Journal article(s); Trial protocol
**Outcome**	Overall survival	**Results**		**Weight**	1

Domain	Signalling question	Response	Comments
**Bias arising from the randomization process**	1.1 Was the allocation sequence random?	N	The allocation sequence was random for WBRT and no-WBRT. Therefore, the trial was not randomized for radiosurgery vs surgery, determining a high risk of bias for this domain.
	1.2 Was the allocation sequence concealed until participants were enrolled and assigned to interventions?		
	1.3 Did baseline differences between intervention groups suggest a problem with the randomization process?		
	**Risk of bias judgement**	**High**	
**Bias due to deviations from intended interventions**	2.1.Were participants aware of their assigned intervention during the trial?	Y	
	2.2.Were carers and people delivering the interventions aware of participants' assigned intervention during the trial?	Y	
	2.3. If Y/PY/NI to 2.1 or 2.2: Were there deviations from the intended intervention that arose because of the experimental context?	PN	
	2.4 If Y/PY to 2.3: Were these deviations likely to have affected the outcome?	NA	
	2.5. If Y/PY/NI to 2.4: Were these deviations from intended intervention balanced between groups?	NA	
	2.6 Was an appropriate analysis used to estimate the effect of assignment to intervention?	Y	
	2.7 If N/PN/NI to 2.6: Was there potential for a substantial impact (on the result) of the failure to analyse participants in the group to which they were randomized?	NA	
	**Risk of bias judgement**	**Low**	
**Bias due to missing outcome data**	3.1 Were data for this outcome available for all, or nearly all, participants randomized?	N	
	3.2 If N/PN/NI to 3.1: Is there evidence that result was not biased by missing outcome data?	PY	
	3.3 If N/PN to 3.2: Could missingness in the outcome depend on its true value?	NA	
	3.4 If Y/PY/NI to 3.3: Is it likely that missingness in the outcome depended on its true value?	NA	
	**Risk of bias judgement**	**Low**	
**Bias in measurement of the outcome**	4.1 Was the method of measuring the outcome inappropriate?	N	
	4.2 Could measurement or ascertainment of the outcome have differed between intervention groups?	N	

	4.3 Were outcome assessors aware of the intervention received by study participants?	Y	
	4.4 If Y/PY/NI to 4.3: Could assessment of the outcome have been influenced by knowledge of intervention received?	PN	
	4.5 If Y/PY/NI to 4.4: Is it likely that assessment of the outcome was influenced by knowledge of intervention received?	NA	
	**Risk of bias judgement**	**Low**	
**Bias in selection of the reported result**	5.1 Were the data that produced this result analysed in accordance with a pre-specified analysis plan that was finalized before unblinded outcome data were available for analysis?	Y	
	5.2 ... multiple eligible outcome measurements (e.g. scales, definitions, time points) within the outcome domain?	N	
	5.3 ... multiple eligible analyses of the data?	N	
	**Risk of bias judgement**	**Low**	
**Overall bias**	**Risk of bias judgement**	**High**	

**Unique ID**	2	**Study ID**	N/A	**Assessor**	L.T. and G.F.
**Ref or Label**	Muacevic et al.	**Aim**	assignment to intervention (the 'intention-to-treat' effect)		
**Experimental**	S + WBRT	**Comparator**	RS	**Source**	Journal article(s)
**Outcome**	Overall survival	**Results**		**Weight**	1

Domain	Signalling question	Response	Comments
**Bias arising from the randomization process**	1.1 Was the allocation sequence random?	Y	
	1.2 Was the allocation sequence concealed until participants were enrolled and assigned to interventions?	Y	
	1.3 Did baseline differences between intervention groups suggest a problem with the randomization process?	N	
	**Risk of bias judgement**	**Low**	
**Bias due to deviations from intended interventions**	2.1.Were participants aware of their assigned intervention during the trial?	Y	
	2.2.Were carers and people delivering the interventions aware of participants' assigned intervention during the trial?	PY	
	2.3. If Y/PY/NI to 2.1 or 2.2: Were there deviations from the intended intervention that arose because of the experimental context?	PN	
	2.4 If Y/PY to 2.3: Were these deviations likely to have affected the outcome?	NA	
	2.5. If Y/PY/NI to 2.4: Were these deviations from intended intervention balanced between groups?	NA	
	2.6 Was an appropriate analysis used to estimate the effect of assignment to intervention?	PY	
	2.7 If N/PN/NI to 2.6: Was there potential for a substantial impact (on the result) of the failure to analyse participants in the group to which they were randomized?	NA	
	**Risk of bias judgement**	**Low**	
**Bias due to missing outcome data**	3.1 Were data for this outcome available for all, or nearly all, participants randomized?	PN	
	3.2 If N/PN/NI to 3.1: Is there evidence that result was not biased by missing outcome data?	PN	
	3.3 If N/PN to 3.2: Could missingness in the outcome depend on its true value?	PN	
	3.4 If Y/PY/NI to 3.3: Is it likely that missingness in the outcome depended on its true value?	NA	
	**Risk of bias judgement**	**Low**	
**Bias in measurement of the outcome**	4.1 Was the method of measuring the outcome inappropriate?	N	
	4.2 Could measurement or ascertainment of the outcome have differed between intervention groups?	N	

	4.3 Were outcome assessors aware of the intervention received by study participants?	Y	
	4.4 If Y/PY/NI to 4.3: Could assessment of the outcome have been influenced by knowledge of intervention received?	PN	
	4.5 If Y/PY/NI to 4.4: Is it likely that assessment of the outcome was influenced by knowledge of intervention received?	NA	
	**Risk of bias judgement**	**Low**	
**Bias in selection of the reported result**	5.1 Were the data that produced this result analysed in accordance with a pre-specified analysis plan that was finalized before unblinded outcome data were available for analysis?	N	
	5.2 ... multiple eligible outcome measurements (e.g. scales, definitions, time points) within the outcome domain?	PN	
	5.3 ... multiple eligible analyses of the data?	N	
	**Risk of bias judgement**	**Some concerns**	
**Overall bias**	**Risk of bias judgement**	**Some concerns**	

**Unique ID**	3	**Study ID**	NCT00124761	**Assessor**	L.T. and G.F.
**Ref or Label**	Roos et al.	**Aim**	assignment to intervention (the 'intention-to-treat' effect)		
**Experimental**	S + WBRT	**Comparator**	RS + WBRT	**Source**	Journal article(s); Trial protocol
**Outcome**	Overall survival	**Results**		**Weight**	1

Domain	Signalling question	Response	Comments
**Bias arising from the randomization process**	1.1 Was the allocation sequence random?	Y	
	1.2 Was the allocation sequence concealed until participants were enrolled and assigned to interventions?	Y	
	1.3 Did baseline differences between intervention groups suggest a problem with the randomization process?	N	
	**Risk of bias judgement**	**Low**	
**Bias due to deviations from intended interventions**	2.1.Were participants aware of their assigned intervention during the trial?	Y	
	2.2.Were carers and people delivering the interventions aware of participants' assigned intervention during the trial?	Y	

	2.3. If Y/PY/NI to 2.1 or 2.2: Were there deviations from the intended intervention that arose because of the experimental context?	PN	
	2.4 If Y/PY to 2.3: Were these deviations likely to have affected the outcome?	NA	
	2.5. If Y/PY/NI to 2.4: Were these deviations from intended intervention balanced between groups?	NA	
	2.6 Was an appropriate analysis used to estimate the effect of assignment to intervention?	PY	
	2.7 If N/PN/NI to 2.6: Was there potential for a substantial impact (on the result) of the failure to analyse participants in the group to which they were randomized?	NA	
	**Risk of bias judgement**	**Low**	
**Bias due to missing outcome data**	3.1 Were data for this outcome available for all, or nearly all, participants randomized?	N	
	3.2 If N/PN/NI to 3.1: Is there evidence that result was not biased by missing outcome data?	PN	
	3.3 If N/PN to 3.2: Could missingness in the outcome depend on its true value?	PY	
	3.4 If Y/PY/NI to 3.3: Is it likely that missingness in the outcome depended on its true value?	N	
	**Risk of bias judgement**	**Some concerns**	
**Bias in measurement of the outcome**	4.1 Was the method of measuring the outcome inappropriate?	PN	
	4.2 Could measurement or ascertainment of the outcome have differed between intervention groups?	PN	
	4.3 Were outcome assessors aware of the intervention received by study participants?	Y	
	4.4 If Y/PY/NI to 4.3: Could assessment of the outcome have been influenced by knowledge of intervention received?	PN	
	4.5 If Y/PY/NI to 4.4: Is it likely that assessment of the outcome was influenced by knowledge of intervention received?	NA	
	**Risk of bias judgement**	**Low**	
**Bias in selection of the reported result**	5.1 Were the data that produced this result analysed in accordance with a pre-specified analysis plan that was finalized before unblinded outcome data were available for analysis?	Y	
	5.2 ... multiple eligible outcome measurements (e.g. scales, definitions, time points) within the outcome domain?	N	
	5.3 ... multiple eligible analyses of the data?	N	
	**Risk of bias judgement**	**Low**	
**Overall bias**	**Risk of bias judgement**	**Some concerns**	
