# Supplementary material for: Surgery vs. Radiosurgery for Patients with Localized Metastatic Brain Disease: A Systematic Review with Meta-Analysis of Randomized Controlled Trials
Source: Cancers (Basel). 2023 Jul 26;15(15):3802. doi: 10.3390/cancers15153802 (PMC10417431; doi:10.3390/cancers15153802)
Supplement: Supplementary file 1 [file cancers-15-03802-s001.zip › Table S2.pdf]

Table S2. Radiosurgery compared to Surgery for patients with localized metastatic brain disease

Certainty assessment							Effect				Certainty
№ of studies	Study design	Risk of bias	Inconsistency	Indirectness	Imprecision	Other considerations	Relative (95% CI)				
**Overall Survival**											
3	randomised trials	serious[a]	not serious	serious[b]	serious[c]	none	HR = 1.01 (CIs: 0.44; 2.32)				⊕◯◯◯ Very low
**Death Rate at 1 year**											
3	randomised trials	serious[a]	not serious	serious[b]	serious[c]	none	101/196 (51.5%)	78/157 (49.7%)	**RR 1.03** (0.84 to 1.27)	**15 more per 1,000** (from 79 fewer to 134 more)	⊕◯◯◯ Very low
**Death Rate at 2 years**											
3	randomised trials	serious[a]	not serious	serious[b]	serious[c]	none	144/196 (73.5%)	107/157 (68.2%)	**RR 1.01** (0.61 to 1.68)	**7 more per 1,000** (from 266 fewer to 463 more)	⊕◯◯◯ Very low
**Local Brain Progression Free Survival**											
2	randomised trials	serious[a]	not serious	serious[b]	serious[c]	none	HR = 0.90 (CIs: 0.58; 1.41)				⊕◯◯◯ Very low
**Local Brain Recurrence Rate at 1 year**											
2	randomised trials	serious[a]	serious[d]	serious[b]	serious[c]	none	35/185 (18.9%)	50/147 (34.0%)	**RR 0.31** (0.04 to 2.42)	**235 fewer per 1,000** (from 327 fewer to 483 more)	⊕◯◯◯ Very low
**Local Brain Recurrence Rate at 2 years**											
2	randomised trials	serious[a]	not serious	serious[b]	serious[c]	none	48/185 (25.9%)	57/147 (38.8%)	**RR 0.64** (0.04 to 10.60)	**140 fewer per 1,000** (from 372 fewer to 1,000 more)	⊕◯◯◯ Very low
**Local Brain Progression Free Survival for radiosurgery alone vs surgical resection + WBRT**											
2	randomised trials	serious[a]	not serious	not serious	serious[c]	none	HR = 1.32 (CIs: 0.69; 2.52)				⊕⊕◯◯ Low

CI: confidence interval; **HR:** hazard ratio; **RR:** risk ratio

Explanations

a. Due to lack of randomization for surgery and radiosurgery during patient allocation in Churilla et al.
b. Due to different employment of WBRT as co-intervention among the included RCTs

c. Due to underpowering small sample size
d. Due to serious heterogeneity
